# Function and Evolutionary Origin of Unicellular Camera-Type Eye Structure

**DOI:** 10.1371/journal.pone.0118415

**Published:** 2015-03-03

**Authors:** Shiho Hayakawa, Yasuharu Takaku, Jung Shan Hwang, Takeo Horiguchi, Hiroshi Suga, Walter Gehring, Kazuho Ikeo, Takashi Gojobori

**Affiliations:** 1 CIB-DDBJ, National Institute of Genetics, Mishima, Japan; 2 Department of Natural History Sciences, Faculty of Science, Hokkaido University, Sapporo, Japan; 3 Department of Cell Biology Biozentrum, University of Basel, Basel, Switzerland; 4 Department of Life Sciences, National Cheng Kung University, Tainan, Taiwan; 5 CBRC/BESE, KAUST, Thuwal, 23955-6900, Kingdom of Saudi Arabia; Institut Pasteur, FRANCE

## Abstract

The ocelloid is an extraordinary eyespot organelle found only in the dinoflagellate family Warnowiaceae. It contains retina- and lens-like structures called the retinal body and the hyalosome. The ocelloid has been an evolutionary enigma because of its remarkable resemblance to the multicellular camera-type eye. To determine if the ocelloid is functionally photoreceptive, we investigated the warnowiid dinoflagellate *Erythropsidinium*. Here, we show that the morphology of the retinal body changed depending on different illumination conditions and the hyalosome manifests the refractile nature. Identifying a rhodopsin gene fragment in *Erythropsidinium* ESTs that is expressed in the retinal body by *in situ* hybridization, we also show that ocelloids are actually light sensitive photoreceptors. The rhodopsin gene identified is most closely related to bacterial rhodopsins. Taken together, we suggest that the ocelloid is an intracellular camera-type eye, which might be originated from endosymbiotic origin.

## Introduction

The evolution of the eye has been of special interest in discussing the origin of complex organs. Charles Darwin called eyes an example of “organs of extreme perfection and complication”. He was quite convinced of his hypothesis that even such a perfect and complex eye could be formed by natural selection[1]. To examine Darwin’s hypothesis, the evolutionary transition of eye form has been studied in various systems, e.g., the pit eyes of worms, and mollusks, and the complex camera-type eyes of vertebrates. However, those studies are restricted to eumetazoan animals[2].

Diverse types of photoreceptive organs have been observed at every level of structural design and ontogenetic origin. In spite of such enormous diversity, an eye can be simply defined as a photoreceptor shielded on one side by pigment which allows the detection of a source of light and its direction[3]. Thus, even a single cell which contains both photo- and shading pigments, as found in some flatworms and algae, fits this definition[3]. Based on this simple definition of eyes, Jékely argued that, although a stigma/eyespot is not always necessary for phototaxis, it increases the sensitivity and ability to detect differences in light intensity[3]. Because they exhibit a variety of eyespots, protists are an excellent starting point to look for an ancestral form of photoreceptive organ or eye.

Dinoflagellates (division Dinophyta, class Dinophyceae) are a group of unicellular protists in marine and fresh waters[4], which have multiple types of eyespots[5,6]. The Warnowiaceae, a family that consists of three heterotrophic genera (*Warnowia*, *Erythropsidinium* and *Nematodinium*), have complex eyespots called “ocelloids”, which are remarkably similar to camera-type eyes[7]. Greuet showed that the ocelloid in warnowiids is a highly developed organelle having cornea-, lens-, and pigment cup-like structures[8]. Greuet also speculated that the ocelloid was a degenerate plastid based on the idea that the presumed ancestor of the Warnowiaceae was photosynthetic[9,10]. However, his speculation has never been substantiated because the physiology and function of the ocelloids are still unknown. Francis determined the reflective index of lens-like hyalosome of *Nematodinium* although he did not explicitly show that the ocelloid was photoreceptive[11]. Neither of these studies examined of molecular composition or photoreceptive functionality of the ocelloid.

With the aim of elucidating the evolutionary origin of camera-type eye, we have investigated the idea that the ocelloid of Warnowiaceae is structurally and functionally an ocular organelle. Because we are currently unable to culture Warnowiaceae, we investigated individual cells that were freshly isolated from marine plankton samples. In the present study, we have focused on the ocelloid of *Erythropsidinium* spp. for the following two reasons: (i) We can regularly collect a relatively large number of specimens of this species from coastal water in Japan and (ii) the ocelloid of *Erythropsidinium* is larger in size than in the other two genera of warnowiids[7], making it easier to handle experimentally.

Our results demonstrate that the ocelloid changes its structure in response to changing illumination and that the retinal body is destroyed by strong illumination. Further, we identify a rhodopsin gene expressed in the retinal body and show that it is related to bacterial, not eukaryotic, rhodopsin. Finally we discuss the evolutionary origin of the ocelloid, an intracellular camera-type eye.

## Results

### The ocelloid in *Erythropsidinium* sp.

**Fig 1:**
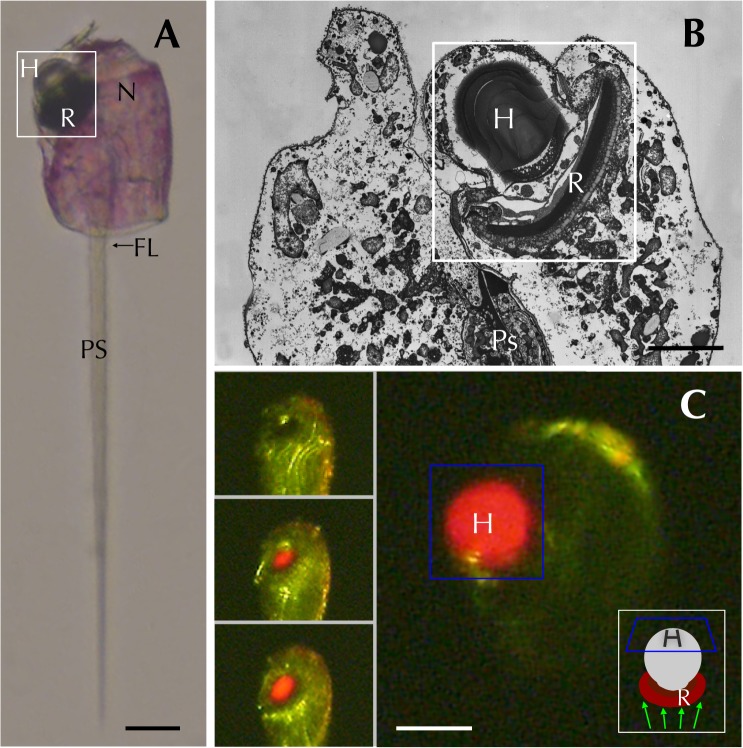
*Erythropsidinium* spp. and its subcellular structure eyespot “ocelloid”. (A) Light micrographs (LM) of *Erythropsidinium* spp. H = hyalosome (crystallin body), R = retinal body, N = nucleus, FL = flagella, PS = piston. (B) Transmission electron micrographs (TEM) of ocelloid. (C) The refractile nature of the hyalosome under fluorescent microscopy. Bars: 20 μm (A, C), 10μm (B). The ocelloid is located at the left side of a cell seen in ventral view according to the orientation proposed by Kofoid and Swezy[12] (Fig. 1A). The nucleus was ellipsoidal and at the opposite side of ocelloid, in the anterior of the cell (Fig. 1B). These indices are consistent with the taxonomic criteria of the type specimen that was identified as *Erythropsidinium agile*. From the serial pictures of autofluorescence in the retinal body (Fig. 1C), lens-effect of the hyalosome can be observed. The front image of the retinal body is larger than side view.

Captured *Erythropsidinium* cells have a large nucleus, one ocelloid, one piston and two flagella (Fig. 1A) [12]. The ocelloid contains a lens-like structure (the hyalosome) and a pigment cup-like component (the retinal body) (Fig. 1B and S1 Fig.). See supporting information for a short video clip that shows the refractive properties of the hyalosome in a live specimen under bright field and fluorescent microscopy (S1 Movie, Fig. 1C). There was some variation in collected specimens in terms of cell size and, for the present experiments, we used medium-sized cells to investigate alterations in morphological and structural features (see Materials and Methods).

### Morphological changes of retinal body in different light conditions

**Fig 2:**
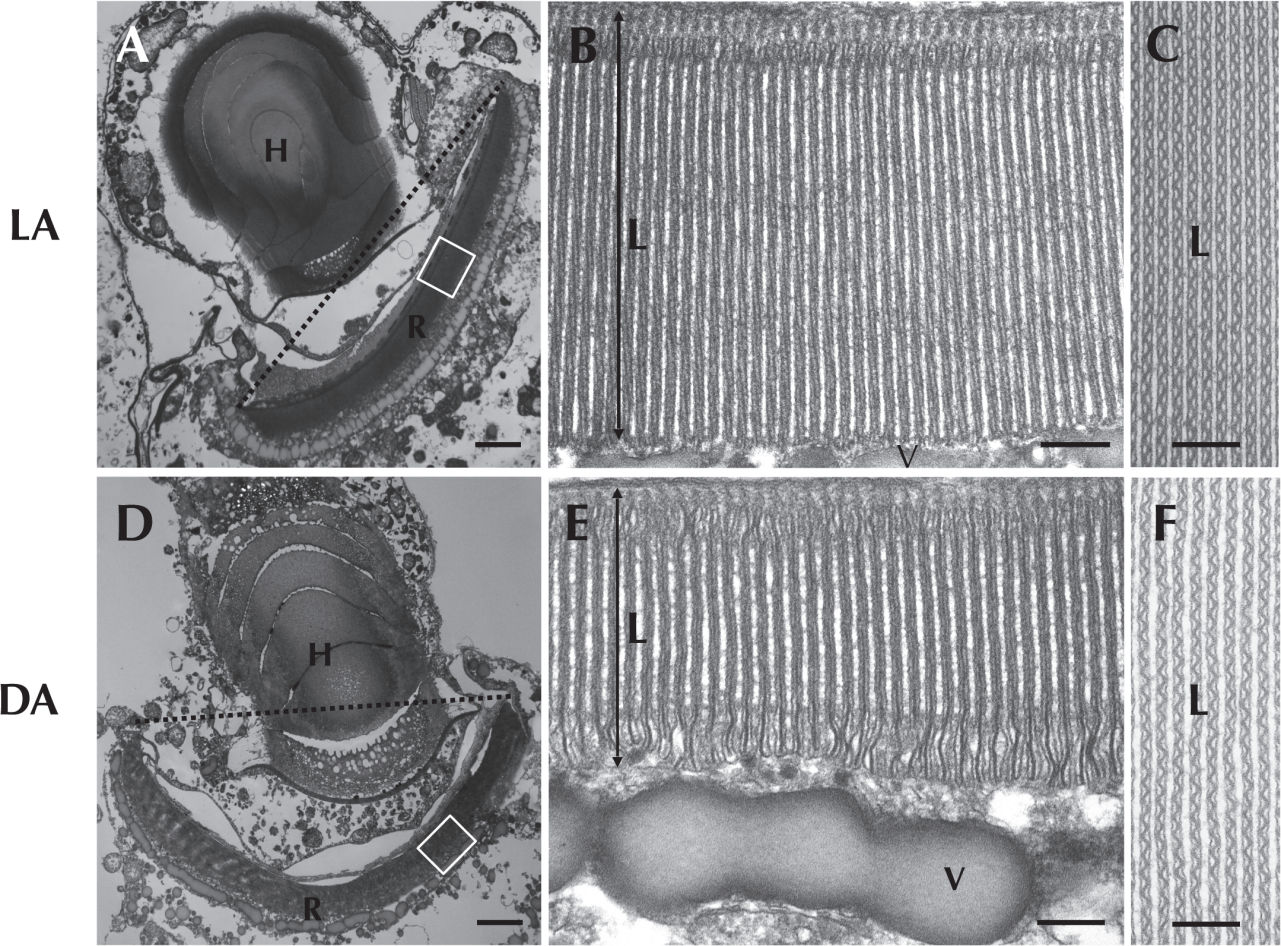
Retinal body of the ocelloid (R) changes its morphology in different light conditions. Light-adapted state (LA: A, B, C) and dark-adapted state (DA: D, E, F) were observed. Enlargement of a longitudinal section of the retinal body (B, E) and cross section (C, F) are shown. L = lamellae, V = vesicular layer. Bars: 2 μm (A, D), 200 nm (B, C, E, F).

To investigate the photoresponse of *Erythropsidinium* cells, we examined ocelloids in two different light conditions (“light- and dark-adapted states”, see Materials and Methods). The retinal body of the ocelloid exhibited significant morphological differences between light- and dark-adapted conditions (Fig. 2A, 2D). Careful observations at higher magnification by TEM, revealed that the retinal body in a light-adapted state had thicker lamellae compared to dark-adapted state (Fig. 2B, 2E).

**Fig 3:**
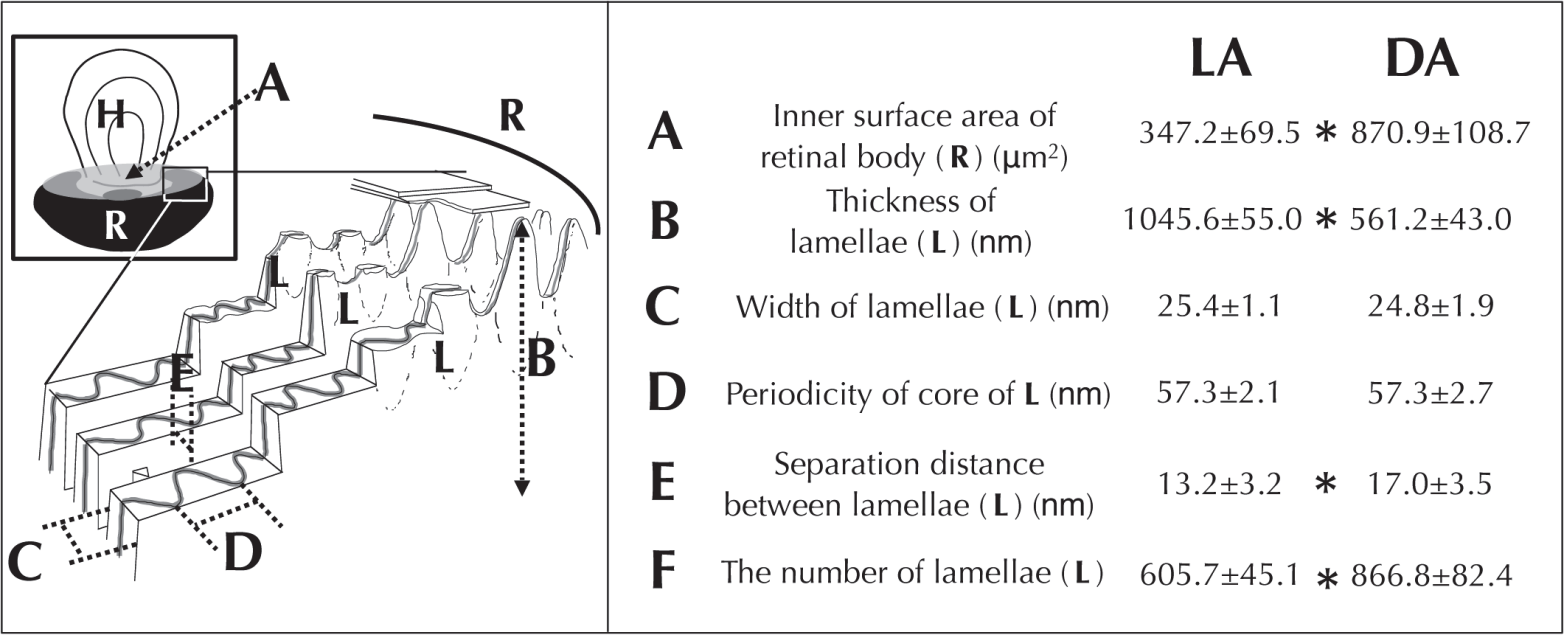
Analysis of morphological differences of the retinal body (R) of the ocelloid. Light-adaptation, LA (Fig. 2B, C and S2 A, B Fig.) versus dark-adaptation, DA (Fig. 2E, F and S2 C, D Fig.). Mean values ± S.D. (N = 25). Significant differences (* P<0.01).

To reveal which part of the retinal body changed its conformation, we measured various morphological features (Fig. 3). The thickness of the lamellae (L) was significantly larger in light-adapted cells (Fig. 2B). Inner surface area of retinal body (a, Fig. 3) and separation distance between lamellae (e, Fig. 3), the number of lamellae (f, Fig. 3) were significantly greater at dark-adapted state. These results show that total inner surface area of the retinal body, which faces the hyalosome, becomes larger in the dark-adapted state compared with light-adapted state (S2A, S2C Fig.). We conclude that the ocelloid has the ability to respond to light-dark conditions.

### The cytoskeleton mediates changes in ocelloid morphology

**Fig 4:**
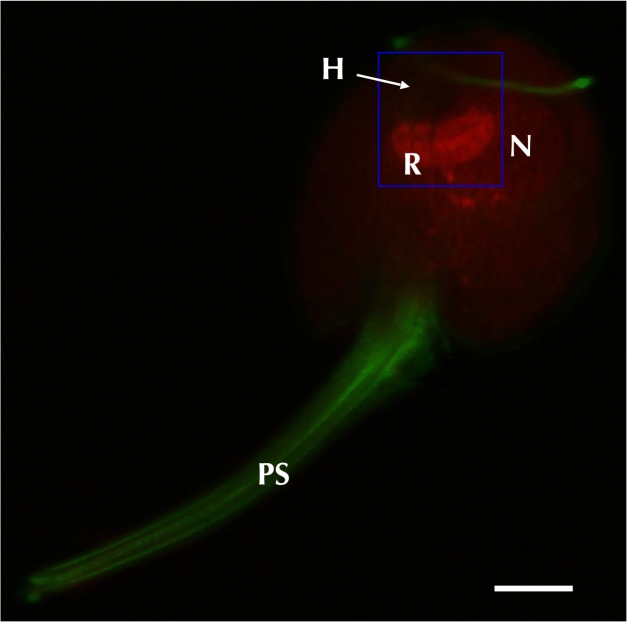
Distribution of cytoskeletal organization was visualized by immunostaining. Bar = 20μm. Tubulin (green) was localized both on the piston (PS) and flagella, and an actin signal (red) was detected on the retinal body of the ocelloid (R).

Next, we investigated the cytoskeleton because it has been shown that photoreceptors in many organisms change conformation in response to light conditions[13–19]. To delineate the distribution of cytoskeletal network, we performed histological analysis by the immuno-staining method. Microtubules were localized in both flagella and piston (Fig. 4). On the other hand, rhodamine-phalloidin staining showed actin filaments distributed onto the retinal body. The hyalosome showed neither signal for actin nor tubulin, suggesting that neither its shape nor spatial position is altered. We compared cells under complete dark conditions with those of light conditions. The distribution of actin filaments was always correlated with morphological change of retinal body, suggesting that it is controlled by actin filaments (S2A–D Fig.). We also examined the effects of cytochalasinB, which inhibits actin polymerization[20]. After treatment with cytochalasinB, filamentous actin was no longer detected in the retinal body or elsewhere in the cell (S2E, F Fig.). The lamellae were also disrupted by the cytochalasinB treatment (S2H, J Fig.). These results indicate that the retinal body changes its conformation under the control of actin filaments in response to changing light conditions.

### Light Damage in vesicular layer

**Fig 5:**
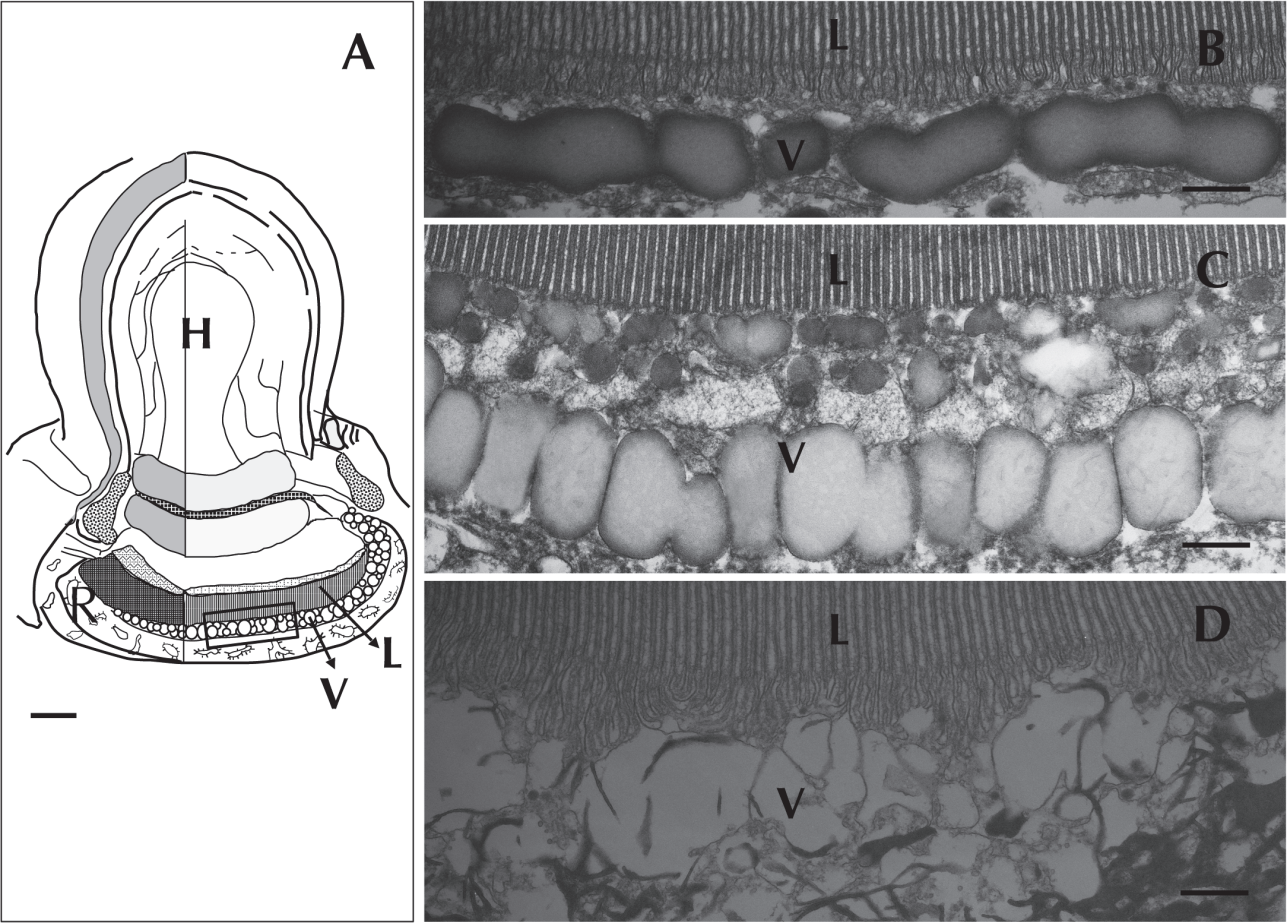
Disruption of vesicular layer (v) in the ocelloid caused by shifting light conditions. Lamellae and ocelloid chamber showed no significant change in morphology (Fig. 5B, C). The fundus area of the retinal body was disrupted (Fig. 5C). Bars: 3 μm (A), 300 nm (B, C).

Strong light, in general, damages photoreceptive cells. To test whether such light-induced damage occurs in the ocelloid, we observed dark adapted cells after exposure to a bright light pulse (see Materials and Methods). We found that the fundus area of the retinal body, named “vesicular layer” by Greuet[10], was disrupted (Fig. 5C). On the other hand, the lamellae (Fig. 5B, C) and ocelloid chamber (not shown) showed no significant difference in morphology after the light pulse. Based on this observation the vesicular layer of the retinal body appears to be photosensitive. Next, we examined whether a plausible photoreceptive molecule is present in the retinal body.

### Localization of mRNA encoding rhodopsin

**Fig 6:**
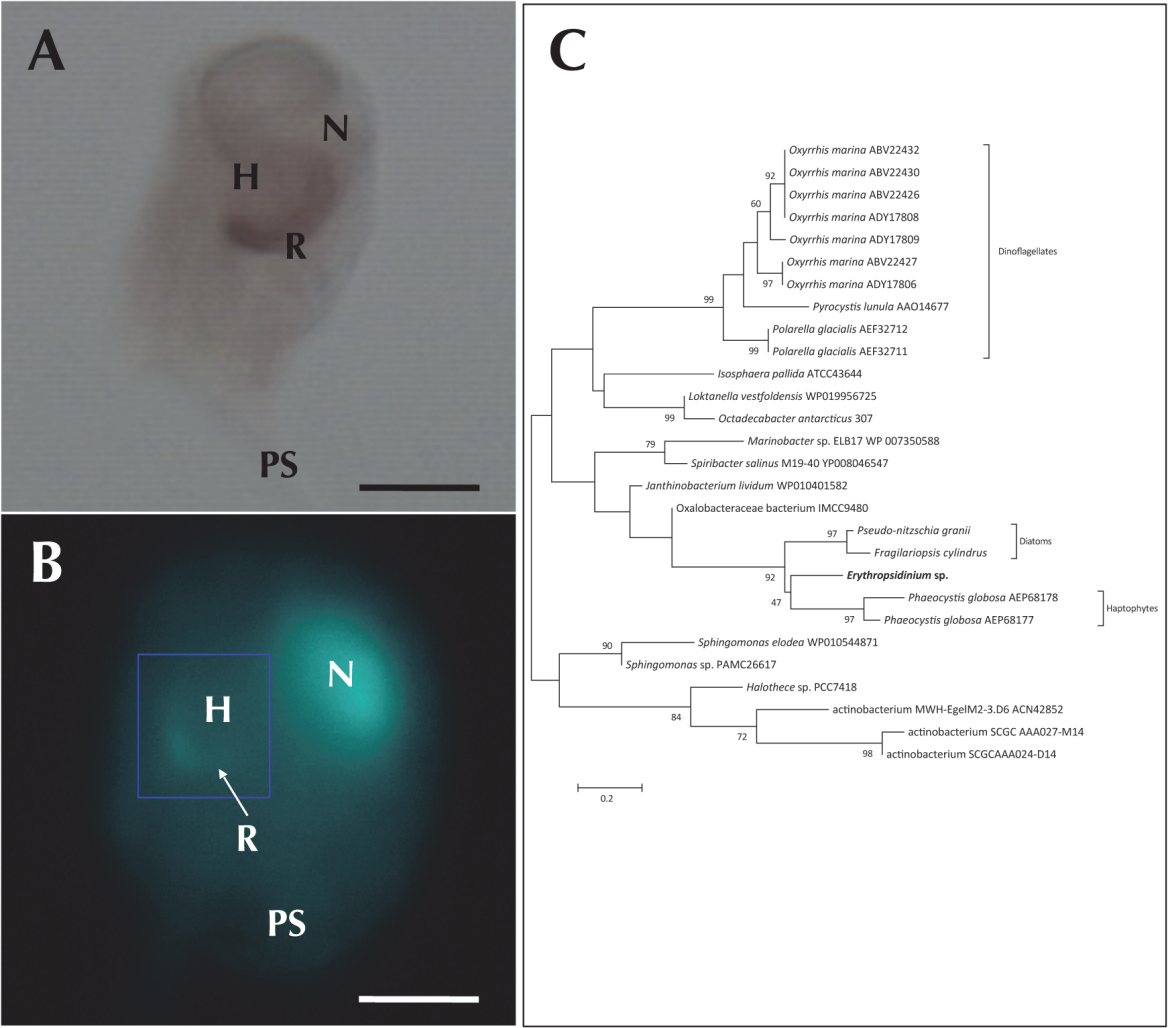
Expression of rhodopsin. Bars: 30μm (A), 50μm (B). (A) *In situ* hybridization using rhodopsin antisense probe. Signal was distributed only in the retinal body. (B) DAPI staining showed signals both on the nucleus (N) and retinal body(R). Hyalosome (H) had no signal. (C) The phylogenetic tree of rhodopsins.

Based on the near universal occurrence of rhodopsin as a photopigment, we search for a rhodopsin gene in *Erythropsidium*. To do this, we isolated mRNA from cells and prepared a cDNA library. We sequenced 1,152 cDNA clones and got 800 ESTs (see Materials and Methods, DDBJ/EMBL/Genbank accession nos: HX969507-HX970306), and searched for possible photoreceptor genes in the cDNA clones. We found one particular clone for a rhodopsin-like gene in the library. We examined the expression pattern of the rhodopsin mRNA by *in situ* hybridization. The probe stained the retinal body (Fig. 6A), suggesting that a rhodopsin-like sequence is expressed there. A sense probe showed no staining.

### Endosymbiotic features of the ocelloid

**Fig 7:**
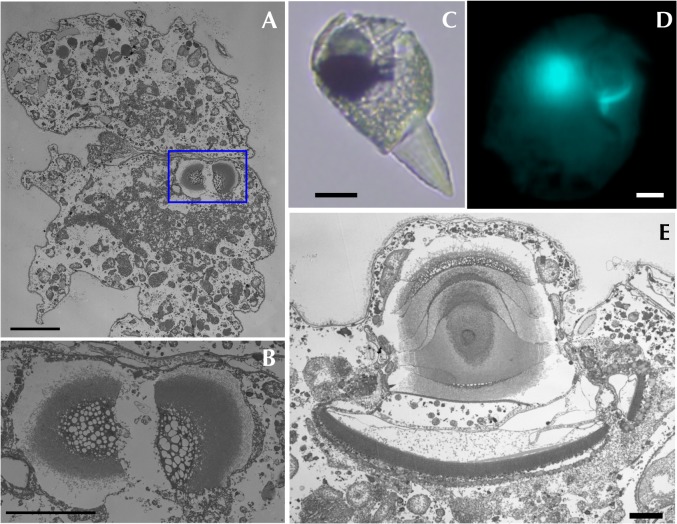
Dividing ocelloid of *Erythropsidinium* spp. Transmission electron micrographs (TEM) of the dividing ocelloid were shown in cross section (A,B) and in longitudinal section (E). (A) The dividing ocelloid (framed in by blue line) is observed in one of the host cell. (B) Partial magnification of ocelloid image. (C, D, E) Dividing retinal body is observed under both light and electron microscopies. Bars: 10 μm (A, D), 5 μm (B), 20μm (C), 2μm (E).

**Fig 8:**
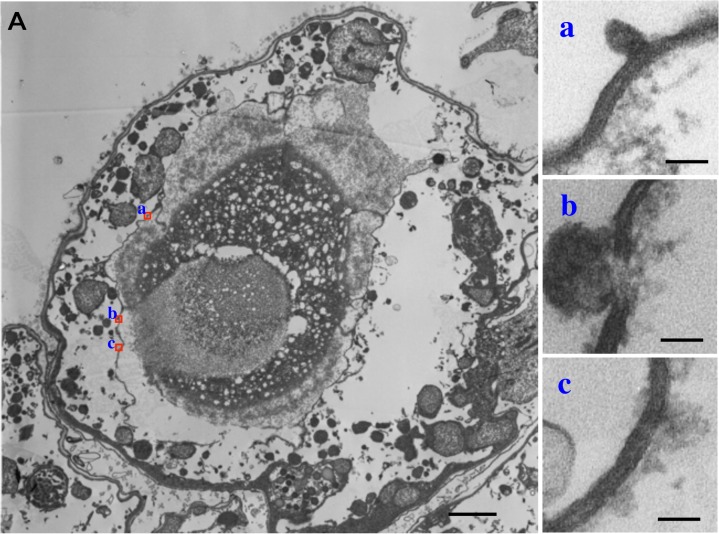
Transmission electron micrographs (TEM) of membranes which are surrounding the ocelloid in *Erythropsidinium* spp. (A) Cross section of the ocelloid. (a-c) Partial magnification of membranes (a, b, c) shown in red square in panel A. Bars: 1μm (A), 40nm (a-c).

It has been suggested that the ocelloid evolved from a chloroplast [10]. If this is the case, it is possible that DNA may be present in the ocelloid. Hence we stained cells with DAPI to localize DNA. In addition to the nucleus, DAPI staining was detected on the retinal-body (Fig. 6B). As we observed the cells using a confocal microscopy with filters to eliminate fluorescence of DAPI/RNA complex, this indicates that nucleotides, such as DNA were present in the retinal body at a site where rhodopsin mRNA was detected by *in situ* hybridization. In some cells, the retinal body was detected in two rings form, indicating that it was dividing. See also Fig. 7D and S2 Movie. Furthermore, by transmission electron microscopy (TEM), we successfully observed that a dividing ocelloid was present in one of dividing host cells (Fig. 7). Moreover, at least two membranes were surrounding the retinal body (Fig. 8). These results showed that the ocelloid has typical features of organelle.

In order to understand the evolutionary origin of the rhodopsin localized in the ocelloid, we conducted a phylogenetic analysis using the sequence of rhodopsin-like gene fragment (83 amino acids, DDBJ/EMBL/Genbank accession no: LC004731). In the ML (same in NJ and ME) tree, the rhodopsin sequence from *Erythropsidinium* branched with rhodopsin genes from haptophytes and diatoms, forming a monophyletic group, though the topology was not strongly supported (Fig. 6C). This group showed a close relationship with bacteriorhodopsin. Thus, it is clear that the rhodopsin-like sequence of *Erythropsidinium* belongs to a bacterial rhodopsin group, the so-called type I. These indicate that the rhodopsin is acquired by horizontal gene transfer.

## Discussion

### The ocelloid is a camera-type eye

The ocelloid has received much attention because of its unique morphological structure. The organelle was described to be much more complex than the simple eyespots of other dinoflagellates [9–11,21]. However, it has not been revealed until now what is the role of the ocelloid and its differentiated structure.

We have shown that the ocelloid contains two elements which are fundamental to a functional camera-type eye; a photoreceptor (the retinal body) and a refractive lens (the hyalosome). Jonasova and Kozmik stated that “Lens-containing (camera-type) eyes can simply be defined as those with an additional refractive element in front of the photoreceptive layer”[22]. This definition clearly supports our idea that the ocelloid of warnowiids is a functional camera-type eye.

### Biological significance of morphological change of the ocelloid

According to our experiments, it is quite likely that the hyalosome functions as a refractive lens to increase the sensitivity to detect light [22] (S3 Fig.). From the results, we concluded that the hyalosome can extend the range of light sensitivity by concentrating dim light.

Regarding the retinal body, the region was shown to be changed in morphology between dark and light-adapted conditions. To support the functional significance of our observation, we utilize a model which has been originally developed to examine optical features of the lens eye of ostracod in fossil record by using morphological metrics data [23] (S3 Fig.). It suggests that the retinal body can receive enough numbers of photons from dim light by using hyalosome to overcome the biochemical activation threshold of the signal transduction system. If it is the case, as a compensatory response, the morphological change of the retinal body under such stronger light (S3 Fig.), also serves to reduce the surface area of the retinal body to avoid a light-induced damage.

Since only weak light penetrates to substantial depths in water, the ocelloid of *Erythropsidinium* seems to be able to change its morphology to exploit weak light more efficiently in a manner analogous to animal eyes. In the recent geographical work by Gómez, most of the specimens of *Erythropsidinium* were collected from depths of less than 90 m. He gave possible interpretation that *Erythropsidinium* tended to inhabit in the illuminated layer where its sophisticated light-receptor is more useful [24]. This observation also supports our finding that the observed morphological change in response to differing light conditions is an adaptation to their ecological niche.

### Actin regulation of morphological change in retinal body

We observed that retinal-body of the ocelloid of *Erythropsidinium* changes its shape under different light conditions (Figs. 2 and 3). Many organisms exploit light as a complex sensory stimulus. For example, plants move their chloroplasts both to enhance photosynthesis and to avoid photo-damage under strong light [17,18,25,26]. Animal eyes exhibit adaptation to dark and light conditions[13–16]. Such morphological changes are regulated by cytoskeletal components, actin and tubulin. Organelle movement in plant cells and organelle translocation in animal cells are regulated by actin filaments and/or microtubulues[14,17,27]. Contraction and elongation of cone cells in fish eyes are regulated by actin and microtubles, respectively[16]. Rhabdom size of octopus[15] and some terrestrial isopods, fly, and crayfish are also controlled by actin filaments[13,14,27]. The morphological change of the ocelloid of *Erythropsidinium* in response to light resembles the alteration in size of rhabdomere cells of some cephalopods and arthropods. Although the former is a subcellular structure whereas the latter is in multicellular system, both are mediated by actin filaments.

### Light damage in retinal body

The structural disruption of the retinal body found in the present study (Fig. 5) showed similar characteristics to light-induced damage studied in arthropod eyes[28] Exposure to a bright light after dark adaptation of the cells induced damage in the “vesicular layer” of the retinal body (Fig. 5C).

It has been shown that the presence of the light sensitive pigment rhodopsin is primarily responsible for acute light-induced damage to mouse retinas[29]. There have also been reports of light-induced morphological damage to photoreceptors in crustaceans, amphipod, arthropods[28] and vertebrates[29]. The phototransduction system in the ocelloid of warnowiid species is not yet known. However, the ultrastructural damage observed in the vesicular layer indicates that at least the vesicular layer is light sensitive. These results suggest that light-induced damage in retinal body of ocelloid might be due to similar mechanisms of photo-damage in animal visual system.

### Rhodopsin in *Erythropsidinium*

In the present study, we observed that mRNA of a rhodopsin was detected only inside the retinal body of the ocelloid. This result bolsters our argument that retinal body is the photosensitive region (Fig. 6A). Gehring showed that the stacked membranes in the retinal body of *Warnowia* had strong birefringence, indicating that the rhodopsin molecules are arranged in a highly ordered paracrystallin fashion[30].

There are two types of rhodopsins, types- I and II. Although they are similar in structure and function, evidence suggests that they evolved independently of one another in bacteria and animals. Both classes are seven-transmembrane proteins that bind to a light-reactive chromophore to mediate responses to light[31]. Type I rhodopsin is found in prokaryotes where it functions as a light-driven proton pump, a light-energy to chemical-energy transducer, and photo-signaling[31,32]. Although some eukaryotic cells are known to have type I rhodopsin, it has been shown to have originated from prokaryotic cells by horizontal gene transfer[32]. On the other hand, Type II rhodopsins are known only in eumetazoan animals and are involved in the photosensitive elements of visual perception and circadian rhythms[33].

The rhodopsin cloned from *Erythropsidinium* belongs to the Type I class. The Type I rhodopsin is known to function as a sensory, regulatory and proton pump in dinoflagellate species[34]. It seems that the rhodopsin in *Erythropsidinium* is closely related to the proton pump group (Fig. 6C). As it is inconsistent with its expression in the retina-like region of the ocelloid, the rhodopsin might be utilized as the photosensitive element in *Erythropsidinium*.

### Endosymbiotic origin of the ocelloid

Greuet speculated (1965) that secondarily acquired chloroplasts may have been transformed into elaborate organelles in some species of dinoflagellates like *Erythropsidinium* and *Warnowia* because extant species lack chloroplasts[10].

We showed that the retinal body was stained by DAPI (Fig. 6B) suggesting that retinal body contains DNA. mRNA of Rhodopsin detected inside the retinal body also raised the possibility that the ocelloid is an endosymbiont with an active genome. It has been reported that rhodopsin in some dinoflagellate species was probably transferred from bacteria by horizontal gene transfer[34,35]. As shown in Fig. 6C, rhodopsin gene cloned from *Erythropsidinium* is closely related to bacterio-type rhodopsin genes of haptophytes and diatoms. It suggests that the rhodopsin gene was originated from bacteria or acquired from diatoms or haptophytes through tertiary endosymbiont.

Therefore, we propose that the ocelloid is an endosymbiont. Endosymbiosis is extremely common in the dinoflagellates. Indeed, recent studies have shown a complex pattern of secondary and tertiary endosymbiosis throughout the dinoflagellate group[36,37].

### Hypothesis for the evolutionary origin of the eye

As discussed above, one of the most important questions is what the evolutionary origin of the ocelloid is. The other is to determine whether the gene components of the ocelloid or some set of genes may have transferred to eumetazoans or not. Gehring (2005) had proposed a hypothesis for the origin of photoreceptor cells in Metazoa [30], which was based on the observation that the eye organelle in flagellates such as *Volvox* or *Chlamydomonas* is located in the chloroplast. He assumed that light perception goes all the way back to cyanobacteria and that it became integrated into eukaryotic cells as chloroplasts. A possible bacterial-like rhodopsin sequence in the ocelloid we found, may support this hypothesis. It will be helpful to determine additional genomic sequences of the endosymbiont for the purpose of determining its evolutionary origin since the ocelloid of Warnowiidae has become a valuable model for the study of the primordial camera-type eye. So far, however, it is difficult to culture *Warnowia* or *Eythropsidinium* and to isolate the organelle from the host, which makes genomic sequencing of the organelle difficult.

Charles Darwin noted that “natural selection has converted the simple apparatus of an optic nerve merely coated with pigment and invested with transparent membrane, into an optical instrument as perfect as is possessed by any member of the great Articulate class [ie,vertebrates]”[1]. There is a concept of “division of labor” which Arendt *et al*. has recently elaborated in their review [38]. They concluded that in order to achieve highly complex metazoan eyes, functional segregation of cell-types may be more important than the acquisition of entirely new cellular functions. In the case of the ocelloid of *Erythropsidinium*, however, a highly elaborate camera-type eye has evolved in a single cell as a subcellular organellar structure.

To address this issue, the elaborate concept has been put forward in which cell-type functional segregation is important in eye evolution of multicellular system[38]. In the unicellular system, as we have shown, even an organelle works as an eye. Thus, intracellular segregation of function may also be important for eye evolution, regardless of involved cell number.

Evolutionary studies of the ocelloid and eyespots in protists may be essential in answering an unsolved question on the evolutionary origin of camera-type eyes that Darwin had raised a long time ago.

## Materials and Methods

### Sample collection

Near-surface plankton samples were collected with a plankton net (mesh size ø35μm) at the docks Shizuura, Suruga Bay (Numazu, Shizuoka) in Japan from August to November of 2007 and 2008. Collected specimens were classified into two types: i) standard type, which are the most commonly observed and ii) cellular division type, which includes some individuals with a median constriction and two pistons, and during observation they divided in two cells (data not shown). Cells with colored particles were also observed. The specimens were distindtively sorted into three categories based on their size. We used the medium-sized type in this study. Immediately after sampling, single cells of *Erythropsidinium* were identified and isolated from the mixed plankton sample by micropipetting, and transferred into filtered seawater (0.2 μm).

Cells used in the light/dark-adaptation study were treated as follows: (1) cells were kept in darkness for one hour (dark-adaptation) or (2) under light conditions for one hour (light-adaptation). Light damage was induced in cells that were kept in darkness at room temperature for 1 hour followed by an exposure to strong white light of 10^7^ photon number for 10 minutes.

For the cytoskeleton study, 2 μM of cytochalasin B (ICN Pharmaceuticals Inc.) was dissolved in 1% dimethyl sulfoxide (DMSO) and added to filtered seawater. For controls, cells were kept in cytochalasin B-free seawater with and without DMSO.

### Visualization of microtubules and actin filaments

Cells were fixed with 4% paraformaldehyde and 0.05% glutaraldehyde in 0.4 M phosphate buffer (pH 7.0) at 4°C for 12 hours, followed by three washes of 10 minutes each in 0.4 M phosphate buffer. To visualize microtubules, a mouse anti-ß-tubulin monoclonal antibody (Sigma) was diluted with 0.2 M phosphate buffer and used at a final dilution of 1:100. Cells were treated with 0.2 M phosphate buffer with 0.1% Triton X-100 at room temperature for 30 minutes followed by incubating in blocking solution (0.2 M phosphate buffer with 0.1% Triton X-100 containing 1% BSA) for 30 minutes.

The cells were then incubated with the antibody solution with 0.1% Triton X-100 for 12 hours in a humid chamber at 4°C, and washed three times in 0.2 M phosphate buffer for 10 minutes each. Subsequently, the samples were incubated with an FITC-conjugated anti-mouse Ig (Amersham) for three hours at 4°C and washed three times with 0.2 M phosphate buffer. The samples were observed under a Carl Zeiss confocal microscope (LSM5 LIVE) with the B filter for FITC.

To visualize actin filaments, rhodamine phalloidin staining was performed. A stock solution of 300 U/ml rhodamine phalloidin (Molecular Probe) was prepared by dissolving the chemical in methanol. It was stored at −20°C as a stock. The cells were stained with rhodamine phalloidin (100-fold dilution of the stock with 0.2 M phosphate buffer) for 60 minutes at 4°C, washed three times with 0.2 M phosphate buffer, and observed using a confocal microscope with the G filter for rhodamine.

### Preparation for transmission electron microscopy

For transmission electron microscopic observations, samples were fixed for 12 hours in 4% glutaraldehyde in 0.1 M cacodylate buffer (pH 7.0) containing 0.25 M sucrose at 4°C, followed by three washes of 10 minutes each in 0.1 M cacodylate buffer (pH 7.0). The cells were then postfixed for 60 minutes in ice-cold 2% OsO_4_ in the same buffer and washed three times for 10 minutes each in ice-cold distilled water. Dehydration through a graded series of ethanol solutions was followed by embedding in an Epon-Araldite mixture. Ultra-thin sections (approximately 60 nm) were made and stained with 2% uranyl acetate followed by 0.4% lead citrate for 5 minutes each. Five serial sections of the widest lamellae of the retinal body were observed for each of 10 cells (50 sections in total) using a JEOL transmission electron microscope (JEM-1010, 80 kV). Statistical analysis of measurement made from the images was carried out with Student’s t test, significance level set at P<0.01.

### Cloning of rhodopsin genes from *Erythropsidinium*

*Erythropsidinium* Cells were manually isolated and washed three times in filtrated sea water and then the whole cells were used to extract total RNA by RNAqueous micro kit (Ambion). Next, RNA was amplified by using a SenseAmp Plus kit (Genisphere). Poly(A+)-tailed RNA was used to make a cDNA library by using a Lambda ZAP-cDNA synthesis and cloning kit (Stratagene). The library had a titer of 3.5 × 10 ^4^ pfu/ml and was amplified to 1.4 × 10 ^7^ pfu/ml; the average insert size was 200 bp with a rage from 400 bp to 1 kbp. After 1152 strings of cDNA sequences were determined by ABI 3730 sequencer (AppliedBiosystems), regions of cloning vectors and ambigious sequences are removed. The resulting 800 ESTs were annotated by BLASTX search (accession numbers HX969507-HX970306).

Rhodopsin-like sequence was detected from BLAST results, and screened from cDNA library by PCR-based method. The partial sequence of rhodopsin gene were obtained (accession number: LC004731) and aligned using T-coffee[39]. The phylogenetic tree was constructed implemented in MEGA version 4 [40].

### *In situ* hybridization

Cells were fixed with 4% paraformaldehyde in 0.4 M phosphate buffer (pH 7.0) and 0.375 M NaCl at 4°C for 12 hours, and followed by three washes of 10 minutes each in 0.4 M phosphate buffer. After dehydration in a graded series of methanol, cells were hydrated gradually and transferred into 0.2 M phosphate buffer. Digoxigenin (DIG)-dUTP-labeled RNA probes were prepared by using a DIG RNA Labeling Kit (Roche Diagnostics, Mannheim, Germany).

Sense and antisense RNA probes were generated from one of the plasmid clones of the cDNA library of *Erythropsidinium* by using one of the two promoters of the pBluescript SK(-) vector (Stratagene). The DIG-labeled RNA probes were added to pre-hybridized cells and incubated overnight at 45°C, and cells were washed in 0.2 M phosphate buffer and blocked for 1 hour at RT, then bound probes were detected with α-DIG-AP. After incubation at 4°C overnight, the cells were washed with 0.2 M phosphate buffer and stained with NBT/BCIP in AP buffer for 30 minutes to overnight at RT.

## Supporting Information

S1 Fig The unique organelle of warnowiids strikingly resemble a camera-type eye. H = hyalosome (crystallin body), R = retinal body/ retina, Cr = Crystallin lens. a. The ocelloid of *Erythropsidinium*. b. Lower eye of *Tripedaria*. c. Ommatidia in fly eye. d. The eye of the marine snail *Murex*. e. A complex camera-type eye in a cuttlefish. f. Vertebrate eye.
(TIF)

S2 Fig Correlation between morphological change of ocelloid and distribution of actin filaments. Bars: 5μm (B, D, F), 50nm (G, H, I, J). A-D. Morphological change of the ocelloid and localization of actin filaments (Red). E, F. After treatment with cytochalasin-B. H, J. Effect of cytochalasin B on a light-adapted cell. G, H. Longitudinal section. I, J. Cross section.
(TIF)

S3 Fig Modeling of the ocelloid of *Erythropsidinium* spp. Simulation using a theoretical morphological model is shown. Ray tracing was simulated to assess the light-gathering abilities of the ocelloid. H = hyalosome, R = retinal body.
(TIF)

S1 Movie Movie of *Erythropsidinium* sp. with autofluorescence in the retinal-body (red colour). Autofluorescence can be observed only from the front and back views but side views.
(MOV)

S2 Movie Serial images of DAPI staining of *Erythropsidinium* cell acquired by a Carl Zeiss confocal microscope (LSM5LIVE). Both the nucleus and the retinal body were detected with a high signal of DAPI. The retinal body was observed as overlapped two rings form, indicating that it was dividing.
(MOV)
