# Supplementary figures and images for: Function and Evolutionary Origin of Unicellular Camera-Type Eye Structure
Source: PLoS One. 2015 Mar 3;10(3):e0118415. doi: 10.1371/journal.pone.0118415 (PMC4348419; doi:10.1371/journal.pone.0118415)

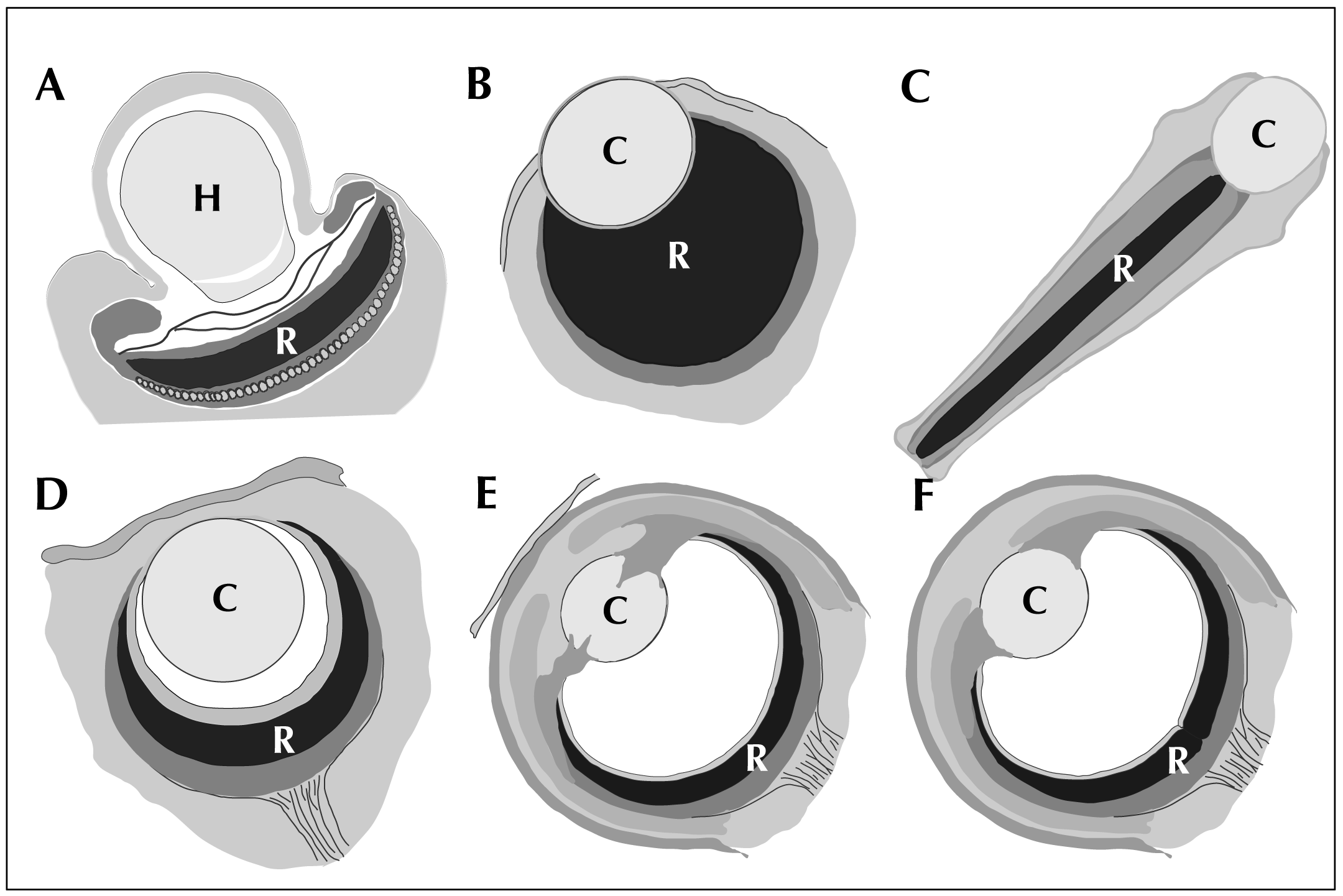

Supplement: S1 Fig — H = hyalosome (crystallin body), R = retinal body/ retina, Cr = Crystallin lens. a. The ocelloid of Erythropsidinium. b. Lower eye of Tripedaria. c. Ommatidia in fly eye. d. The eye of the marine snail Murex. e. A complex camera-type eye in a cuttlefish. f. Vertebrate eye. (TIF) [file pone.0118415.s001.tif]

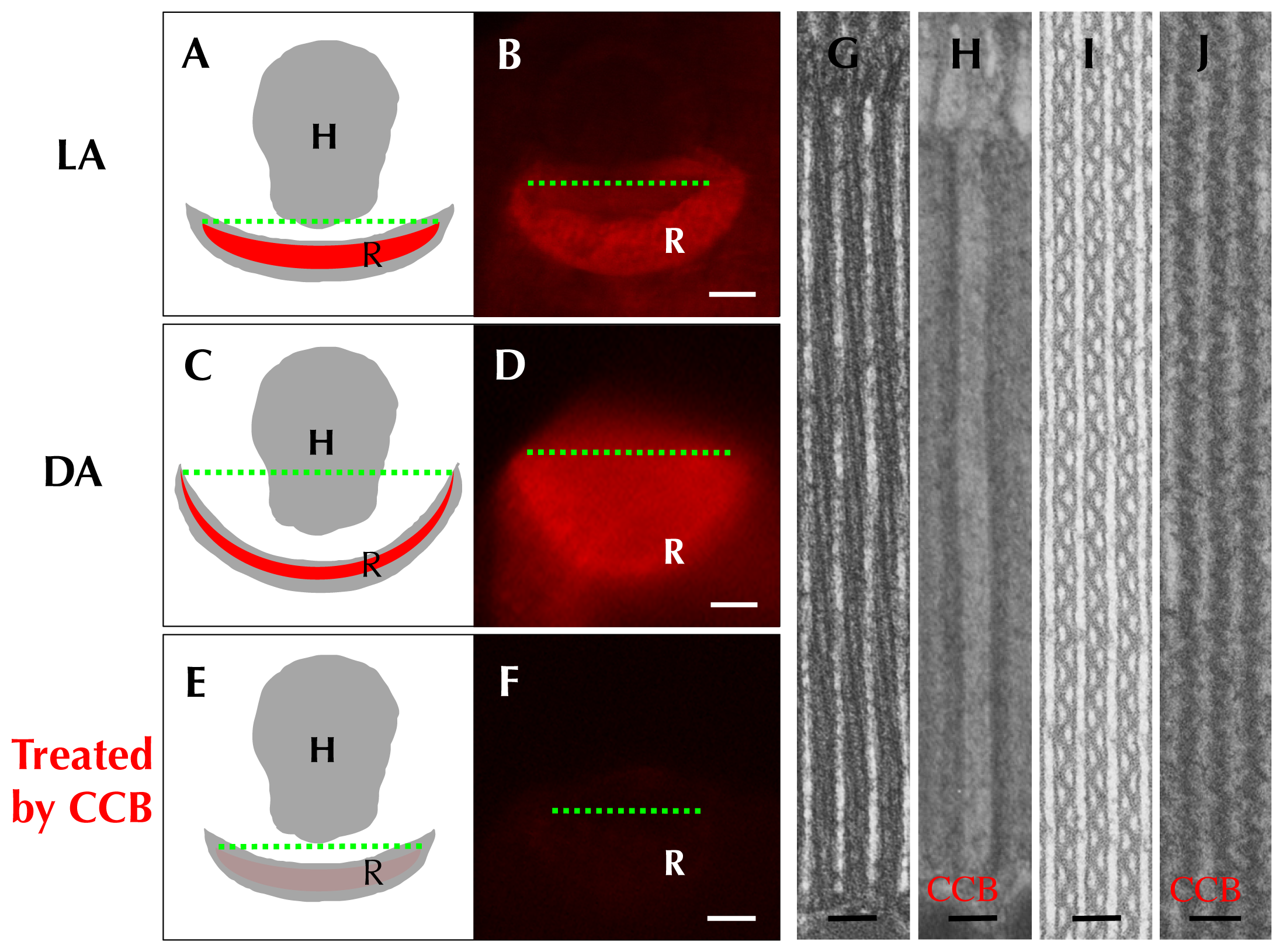

Supplement: S2 Fig — Bars: 5μm (B, D, F), 50nm (G, H, I, J). A-D. Morphological change of the ocelloid and localization of actin filaments (Red). E, F. After treatment with cytochalasin-B. H, J. Effect of cytochalasin B on a light-adapted cell. G, H. Longitudinal section. I, J. Cross section. (TIF) [file pone.0118415.s002.tif]

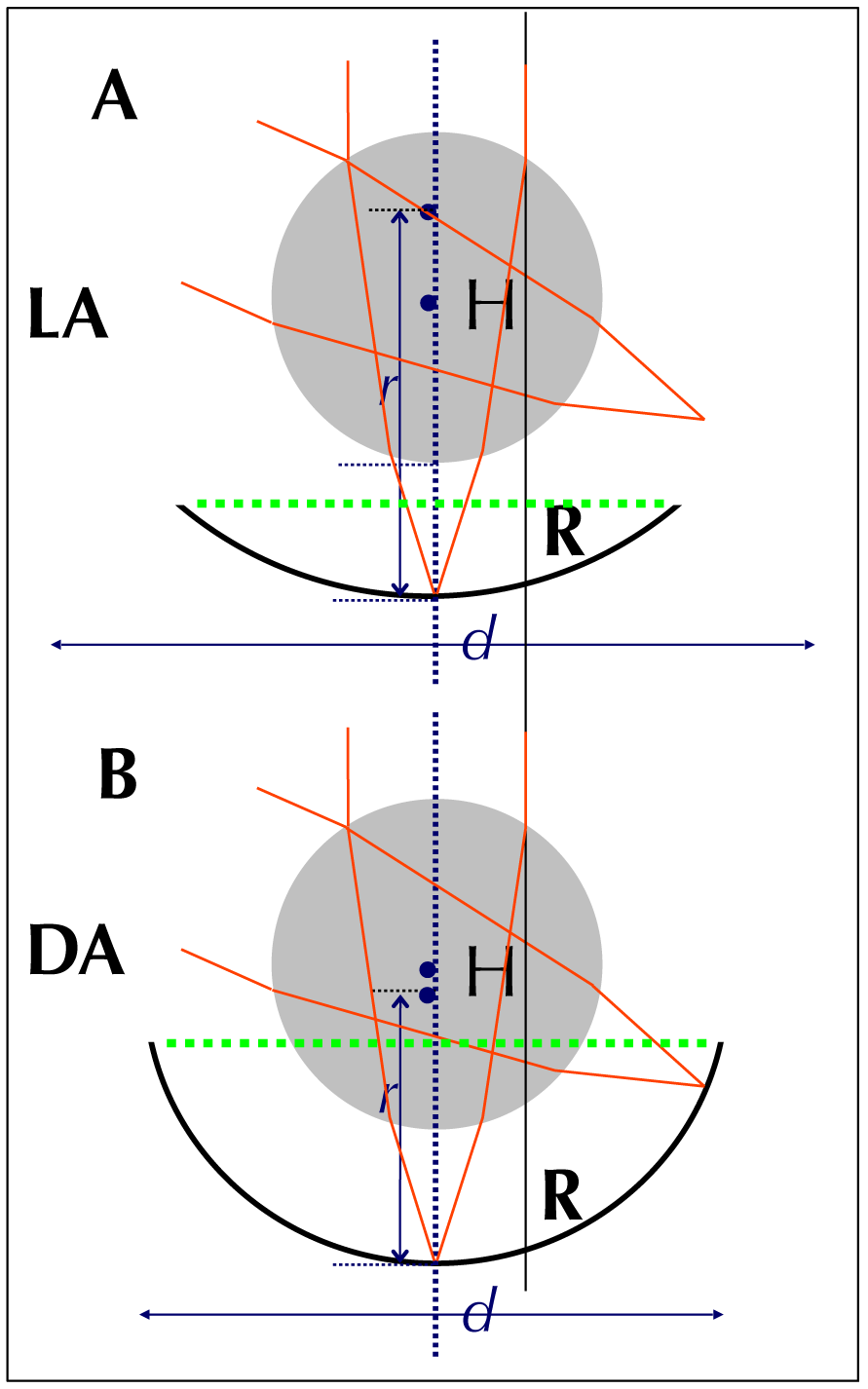

Supplement: S3 Fig — Ray tracing was simulated to assess the light-gathering abilities of the ocelloid. H = hyalosome, R = retinal body. (TIF) [file pone.0118415.s003.tif]
